# Editorial: Mechanisms of Epigenetics and Genetics in Leukemogenesis

**DOI:** 10.3389/fonc.2022.896094

**Published:** 2022-04-07

**Authors:** Yonghui Li, Fei Gao, Shujun Liu

**Affiliations:** ^1^ Central Laboratory, Shenzhen Key Laboratory of Precision Medicine for Hematological Malignancies, Shenzhen University General Hospital, Shenzhen University Health Science Center, Shenzhen, China; ^2^ Shenzhen Branch, Guangdong Laboratory for Lingnan Modern Agriculture, Genome Analysis Laboratory of the Ministry of Agriculture, Agricultural Genomics Institute at Shenzhen, Chinese Academy of Agricultural Sciences, Shenzhen, China; ^3^ The Hormel Institute, University of Minnesota, Austin, MN, United States

**Keywords:** leukemia, epigenetics, genetics, immunotherapy, *n* ^6^-methyladenosine

According to the new cancer statistic report, the incidence and mortality of leukemia rank among the top ten of all cancers (1). Understanding the mechanism of leukemia is vitally important which might help us to identify novel markers and develop novel therapeutic strategies. With the development of science and technology, especially the sequencing technique, a number of studies have depicted the genetic and epigenetic landscape of leukemia (2–4). In the meanwhile, a large scale of databases containing different sequencing data of leukemia have been established and broadly open-accessed, for example, the TCGA database. It has been conclusively shown that genetic and epigenetic abnormalities contribute greatly to the generation, progression, and drug resistance of leukemia. However, the mechanism of leukemia is far from being fully elucidated. The articles in the Research Topic on *Mechanisms of Epigenetics and Genetics in Leukemogenesis* explored both genetic and epigenetic mechanisms in leukemia generation.

Genetic alterations to genes involved in hematopoiesis, tumor suppressor genes, and oncogenes can result in dramatic gene expression changes leading to leukemia. Zhang et al. showed that RUNX3 is highly expressed in acute myeloid leukemia (AML) cells. Further study revealed RUNX3 knockdown inhibits AML progression by altering the expression of genes involved in DNA damage and apoptosis. Su et al. reviewed CEBPA mutation in leukemia including current progress and future directions. Patients with different subtypes of CEBPA mutations showed different clinical features and different sensitivity to chemotherapy, which can be useful for optimizing the clinical management of AML patients with CEBPA mutations. Shi et al. showed that high CENPE expression is correlated with chemoresistance, while knockdown of CENPE expression *in vitro* suppresses the proliferation of myeloid leukemia cells and reverses the cytarabine (Ara-C) chemoresistance.

Since the beginning of the 21st century, epigenetics has entered a period of rapid development, especially in the field of biology and medicine. Examples of epigenetic modifications mainly include DNA methylation, histone modifications, chromatin remodeling, and non-coding RNAs. ASH1L is a histone methyltransferase that is essential in the generation and maintenance of MLL-AF9 leukemia. Aljazi et al. reported that ASH1L binds to the promoters and modifies the local histone H3K36me2 levels of MLL-AF9 target genes including *Hoxa9* and *Hoxa10*. SET8 regulates the histone H4 monomethylation at Lys 20 (H4K20me1), which is highly expressed in AML and associated with poor prognosis (Xu et al.). Targeting SET8 by LukS-PV induces apoptosis in leukemia. Besides methylation, histone acetylation plays a vital role in leukemogenesis. Zhang et al. made an elaborate review on the roles of histone deacetylases (HDAC) in AML with fusion proteins. lncRNA PPM1A-AS is highly expressed in T-cell acute lymphoblastic leukemia (T-ALL) and regulates genes in multiple signaling pathways. Li et al. confirmed that PPM1A-AS acts as an oncogene in T-ALL by promoting cell proliferation and inhibiting cell apoptosis. Another type of epigenetically regulated genes are those associated with immunity. According to the study from Xiao et al., Intercellular Adhesion Molecule‐1 (ICAM-1), a crucial factor in tumor immunity, is epigenetically silenced by DNA methylation. The use of decitabine restores ICAM-1 expression and inhibits AML immune escape from NK cells. This study suggests that combining hypomethylating agent decitabine and NK cell infusion may be a potentially effective strategy in AML treatment.

Accumulated study of genetics and epigenetics in leukemogenesis facilitates the identification of possible novel biomarkers and the study of new targeted drugs, including the abovementioned CENPE (Shi et al.), ASH1L **(** Aljazi et al.), and SET8 **(** Xu et al.). In addition, HDAC inhibitors and hypomethylating agents (azacytidine, decitabine) have been widely used in clinical practice and the effect has been widely proved (5–7). In our Research Topic, Yin et al. reported a phase II clinical trial using a regimen combining chemotherapy, HDAC inhibitor, and hypomethylating agent in patients with relapsed/refractory AML. The completed remission (CR) rate is 42.9%, which suggests the double epigenetic priming regimen has good antileukemia activity. It indicates that a better understanding of the genetic and epigenetic mechanism of leukemogenesis has recently begun to increasingly influence the clinical decisions from diagnosis and risk stratification to individual therapeutic intervention.

In summary, the original articles, reviews, and clinical trials collected in this Research Topic represent an invaluable resource of insights on mechanisms of genetics and epigenetics in leukemogenesis. However, more studies, particularly on the interaction between genetics and epigenetics are needed to fully understand the mechanism of leukemogenesis, which will guide future clinical trials and lead to the development of new therapeutic strategies.

## Author Contributions

YL, FG, and SL are co-editors for this Research Topic. All authors contributed to the article and approved the submitted version.

## Funding

YL was supported by National Natural Science Foundation of China (82070161, 81870134, and 81570137) and Beijing Natural Science Foundation (7202186).

## Conflict of Interest

The authors declare that the research was conducted in the absence of any commercial or financial relationships that could be construed as a potential conflict of interest.

## Publisher’s Note

All claims expressed in this article are solely those of the authors and do not necessarily represent those of their affiliated organizations, or those of the publisher, the editors and the reviewers. Any product that may be evaluated in this article, or claim that may be made by its manufacturer, is not guaranteed or endorsed by the publisher.
